# A Neuropsychological Rehabilitation Program for Cognitive Impairment in Psychiatric and Neurological Conditions: A Review That Supports Its Efficacy

**DOI:** 10.1155/2019/4647134

**Published:** 2019-10-21

**Authors:** Ainara Gómez-Gastiasoro, Javier Peña, Naroa Ibarretxe-Bilbao, Olaia Lucas-Jiménez, María Díez-Cirarda, Oiane Rilo, Genoveva Montoya-Murillo, Leire Zubiaurre-Elorza, Natalia Ojeda

**Affiliations:** Department of Methods and Experimental Psychology, Faculty of Psychology and Education, University of Deusto, Avenida de las Universidades, 24, 48007 Bilbao, Biscay, Spain

## Abstract

Neuropsychological rehabilitation has been the focus of much scientific research over the past decades due to its efficacy in different pathologies. Advances in the neuropsychology field have led to improvements and changes in neuropsychological interventions, which in turn have given rise to different approaches and rehabilitation programs. REHACOP is an integrative neuropsychological rehabilitation program designed by specialist neuropsychologists. With an integrated bottom-up and top-down approach, REHACOP includes neurocognition, social cognition, and daily living tasks hierarchically organized on an increasing level of difficulty. Task arrangement is addressed to maximize improvements and transfer effects into participant's daily living. To date, REHACOP has been implemented on different clinical samples such as patients with schizophrenia, multiple sclerosis (MS), and Parkinson's disease (PD). This manuscript presents the efficacy data of REHACOP across these three populations and discusses it in the context of the available literature. Overall, the magnitude of improvements obtained by means of REHACOP ranged from medium to high across samples. These changes were not restricted to specific neurocognitive domains since participants attending the REHACOP program also showed changes in social cognition and daily functioning variables by means of both direct and transfer effects. Results regarding REHACOP's efficacy in psychiatric and neurological conditions have contributed to expanding the existing evidence about the use of structured neuropsychological rehabilitation. In addition, the results obtained after its implementation highlighted the need and importance of designing and implementing integrative neuropsychological rehabilitation programs that are focused not only on cognition *per se* but also on participants' performance in daily living.

## 1. Introduction

**Figure 1:**
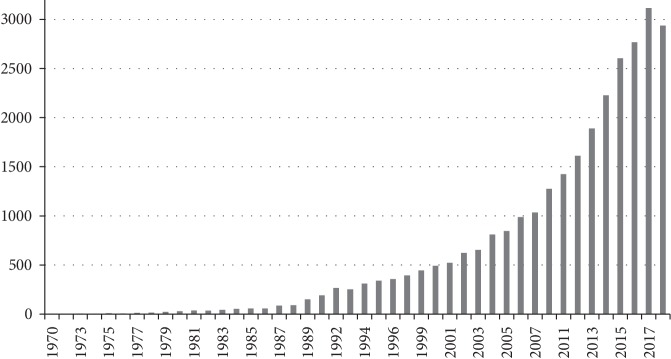
Number of publications including “cognitive rehabilitation” terms in PubMed.

While neuropsychological rehabilitation has been the focus of much scientific research in different disciplines since the late 1970s (see Figure 1), its origins and conceptualization date back to the First and Second World Wars [1–3], and other even remoter roots (Ancient Egypt [4], Paul Broca [5], etc.). As neuropsychology itself, cognitive rehabilitation history is strongly tied to historical medical advances [1–3]. The survival of brain-injured soldiers created the opportunity and the need to work for the recovery of the lost cognitive functions due to focal brain injuries [2]. In light of this, the initial focus of cognitive rehabilitation in its earliest years was traumatic brain injury patients with focal damages. Even at that time (1940s), cognitive rehabilitation was described from an integral approach, involving not only the affected cognitive domain *per se* but also patients' social, functional, and family areas [2]. It was also in that decade when Oliver Zangwill outlined the three principles of cognitive rehabilitation (compensation, substitution, and direct training; [6]) which eventually turned out to be the basis for almost all currently existing cognitive rehabilitation programs.

It was not until the late 1980s and the early 1990s that neuropsychological rehabilitation started to be applied to other neurological conditions, including neurodegenerative diseases such as dementia [7]. This implementation included slight modifications to how neuropsychological rehabilitation was conceptualized and what its aims were, given the course of neurodegenerative diseases. This highlighted the role of cognitive rehabilitation as an instrument for both optimizing and minimizing the extent of a disability, instead of trying to achieve full recovery [7]. From the late 1990s and in the early 21^st^ century, neuropsychological rehabilitation was also implemented in psychiatric patients in an attempt to address those cognitive impairments that could not be treated by either typical or atypical antipsychotics [8–10]. Therefore, neuropsychological rehabilitation principles and techniques have historically shown potential benefits for various brain disorder profiles.

As stated by Wilson [11], neuropsychological rehabilitation has been defined differently over the years, according to the changes and advances in the neuropsychology field. In addition, different concepts related to neuropsychological rehabilitation have also emerged during history. For instance, cognitive rehabilitation is usually broadly defined as a process whereby a brain-injured patient works together with professionals to remediate or alleviate cognitive deficits [3]. A concept that is closely related to neuropsychological rehabilitation is cognitive stimulation, which refers to all those activities that focus on improving general cognitive functioning or some specific domains such as attention, language, or memory [12], with no specific mention to how those activities are organized or structured. Nevertheless, some of those and other terms are usually indistinctly used in the literature, along with other concepts such as cognitive remediation. However, neuropsychological rehabilitation has been proposed as a broader concept than those previously mentioned as it addresses not only cognitive impairments but also those emotional, psychosocial, and behavioral deficits caused by any brain damage in a more or less structured way [3].

### 1.1. The REHACOP Program

**Figure 2:**
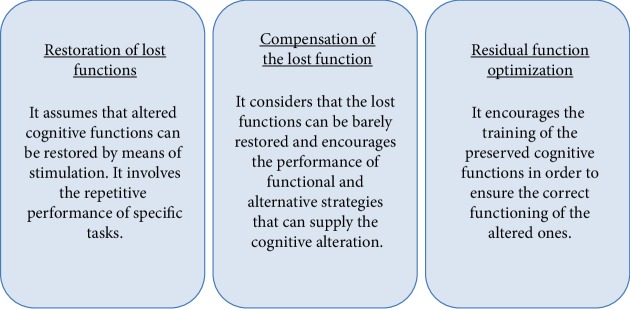
REHACOP's three basic principles [13].

REHACOP (http://rehacop.deusto.es) is an integrative neuropsychological rehabilitation program that was designed by neuropsychology experts at the University of Deusto [13], relying on the knowledge that has been previously generated. The program was originally developed to provide the first available standardized intervention in neuropsychological rehabilitation for Spanish patients with schizophrenia. However, it was later adapted to other clinical populations including neurological conditions such as multiple sclerosis (MS) and Parkinson's disease (PD) [14–17]. Due to the high levels of satisfaction obtained both among patients and therapists, and its efficacy, this manuscript seeks to describe the characteristics of the process involved, as it could be useful for similar interventions elsewhere. REHACOP's specific aims are based on the effective principles of neuropsychological rehabilitation enunciated by Zangwill [6], previously mentioned (Figure 2, [13]).

REHACOP is composed of more than 300 paper-and-pencil tasks using a bottom-up approach (about 85%), and a final integration with top-down tasks (about 15%), following a design that has showed efficacy [18]. This approach highlights the importance of implementing bottom-up approaches in neuropsychological rehabilitation programs in order to have an impact on the basic cognitive processes that have been altered and that could be affecting higher-order functions [19, 20]. Tasks are divided into eight modules, each of which is focused on a specific cognitive domain or area, namely, (a) attention, (b) learning and memory, (c) language, (d) executive functions, (e) social cognition, (f) social skills, (g) activities of daily living, and (h) psychoeducation. The psychoeducation module is focused on the pathology and symptom management. Given that processing speed is a cross-cutting domain, its training takes place along with the first four modules, since a lot of the tasks can be optionally timed. REHACOP is hierarchically organized to provide an order and a bottom-up approach to therapists, so that patients gradually acquire abilities that require building up from the most basic to the most demanding cognitive processes. Both the modules and the tasks included in them are arranged based on an increasing level of difficulty, until it becomes possible for participants to use the skills they have been trained in their real daily life. As the program allows both individual and group intervention, the therapist can recreate or simulate close to real scenarios to train the patient and facilitate later generalization. This specific arrangement means that the program can facilitate the generalization of trained skills to daily living [13].

The REHACOP program includes both a patient and a therapist manual, so all the materials are in a paper-and-pencil format. This allows subjects to be retrained in skills that are closer to those originally acquired across their lifespan. In the therapist's manual, the professional can find explanatory pages that include the name of the task, information about the cognitive domain and subdomain that the training is focused on, the level of difficulty, the specific instructions for the therapist, the specific instructions to be given to the patient, the necessary materials for each task to be performed, and also an answer sheet with the correct answers to all the tasks [13]. This overall structure facilitates adherence to the underlying principles, which is in the best interest of efficacy and scientific replication.

The recommended time for the implementation of REHACOP in terms of time and frequency is five months, three sessions per week, and (at least) 30-minute sessions for individual interventions and (at least) 90-minute sessions for group interventions, in groups of between 6 and 8 patients with one therapist [13]. Group format was selected due to time and human resource constrictions, allowing the REHACOP implementation in more than one individual in each session. However, efficacy has also been enhanced by having shorter interventions, as will be described later in this paper. Although the first version of REHACOP was in Spanish, the program has now been translated into Portuguese and is currently being translated into English, Polish, and Greek.

For schizophrenia [21], MS [14], and PD [15–17] studies that used REHACOP described in the present review, the intervention lasted for 13 weeks and consisted of three group sessions per week. Each session lasted 60 minutes each for the MS and PD groups and 90 minutes each for the schizophrenia group. The sessions' structure for the schizophrenia, MS, and PD studies was designed and implemented as follows: (1) weeks 1 to 4—attention training (sustained, selective, divided, and shifting attention); (2) weeks 5 to 7—learning and memory training; (3) weeks 8 to 10—language training (grammar, syntax, vocabulary, etc.); (4) weeks 11 to 12—executive function training (planning, analogies, etc.); and (5) week 13—social cognition training. Due to the lack of a social cognition module at the time that the two other schizophrenia studies were performed [22, 23], these did not include social cognition training, whereas the rest of the timetable remained the same. In addition, one of the schizophrenia studies [22] included social skills, activities of daily living, and psychoeducation modules. A summary of the implementation's characteristics and main results is described in Table 1.

## 2. Implementation of the REHACOP Program in Schizophrenia, Multiple Sclerosis, and Parkinson's Disease

### 2.1. Schizophrenia

Cognitive impairment has been well described in patients with psychosis and schizophrenia. Their performance has been found to be at least 1 or 1.5 standard deviations below the mean when compared to healthy controls across most of the cognitive domains [24]. However, cognitive deficits are present to a different extent depending on the domain. Some of the most altered domains in patients with schizophrenia are verbal memory, processing speed, language, and nonverbal memory [25–27]. These alterations are present both at the beginning of the disease [26] and at the chronic period [25]. Although to a lesser extent, other cognitive functions such as visuospatial abilities, executive functioning, and working memory are also affected in these patients when their performance is compared to that of normal controls [25, 26]. Furthermore, cognitive deficits have also been described even before the onset of the disease [28, 29] and in naïve patients [30]. Special attention must be paid to basic cognitive processes such as visual or auditory perception, which are also impaired in these patients [31] and might have an important role in the neuropsychological rehabilitation process [19, 20].

Beyond neurocognitive deficits, patients with schizophrenia also show deficits in social cognition [32]. These social cognition alterations seem to be present in all of its four most commonly recognized domains (theory of mind, social perception, emotion perception, and emotion processing) [32]. However, the magnitude of the deficits is greater in the social perception and theory of mind domains [32].

Neurocognition and social cognition deficits have been found to be strongly related to functional alterations in patients with schizophrenia, especially in the case of social cognition [33]. The strongest associations regarding neurocognition have been found between verbal fluency and community functioning and between verbal learning and memory and social behavior in the milieu [33]. When looking at the relationship between social cognition and functionality, the association between theory of mind and community functioning seems to be the most important, followed by the association between emotion processing and social behavior in the milieu [33].

Brain alterations have been also described in patients with schizophrenia. Regarding anatomical alterations, both gray and white matter volume reductions are described in these patients [27], along with abnormalities in white matter integrity [34]. The latter are mainly circumscribed to frontal and temporal areas located in the left hemisphere [34]. Functional brain alterations have also been described both during performance of a task and at resting state [27]. Studies have related the abnormalities mentioned to cognitive performance and symptoms in these patients, highlighting their role in any rehabilitation process.

### 2.2. REHACOP in Schizophrenia

Different studies have supported REHACOP's efficacy in improving not only patients' neurocognition [21–23] but also their social cognition [21], clinical symptoms [21, 22], and functionality [21, 22]. Patients on these studies were randomized to the REHACOP or the active control group which performed occupational activities with the same duration and frequency as the REHACOP group (38 patients in the experimental group vs. 38 patients in the active control group [23], 36 patients in the experimental group vs. 48 patients in the active control group [22], and 52 patients in the experimental group vs. 49 patients in the active control group [21]). Patients with schizophrenia receiving REHACOP intervention have shown neurocognitive improvement in processing speed, verbal memory, verbal fluency, working memory, and executive functions [22, 23], as well as in global neurocognition scores [21]. Effect sizes of these improvements have ranged from medium to high depending on the assessed domain with verbal memory and overall cognition showing the largest effects (*d* = .88 and *η*_*p*_^2^ = .14, respectively) [21, 22]. Among social cognition domains, theory of mind has shown the greatest improvements after implementation (*η*_*p*_^2^ = .15), followed by social perception (*η*_*p*_^2^ = .08) and emotion processing (*η*_*p*_^2^ = .07) [21]. Patients with schizophrenia receiving REHACOP have also shown a decrease in negative (*η*_*p*_^2^ = .08 and *d* = .48) but not in positive symptoms after the intervention [21, 22].

The REHACOP program has also shown efficacy for improving the social functioning of these patients in a wide range of domains, including functional competence, global functioning, and social competence [21, 22]. This has highlighted the presence of both direct and transfer effects in this pathology. Furthermore, in a more specific study focused on the mechanisms through which REHACOP improved functional outcomes in patients with schizophrenia, functional improvements were found to be mediated by changes in cognition [35]. Specifically, functional outcome changes were mediated by processing speed and verbal memory improvements, but not by improvements in social cognition and negative symptoms [35]. The existence of any brain changes after the implementation of the REHACOP has not yet been studied in this population.

### 2.3. Multiple Sclerosis

Cognitive impairment is also present in neurological conditions such as MS [36–38]. Cognitive alterations in patients with this pathology include deficits in attention, executive functioning, long-term visual and verbal memory, and visuoconstructive abilities, as well as global cognition [36–38]. Processing speed is also particularly impaired in patients with MS, affecting performance across all other cognitive domains [36]. Deficits have also been described in relation to social cognition, as MS patients have been shown to have difficulty in performing tasks involving theory of mind or emotion processing [39]. As in schizophrenia, cognitive decline has been related to a decreased functional outcome among MS patients. Specifically, the presence of cognitive deficits is related not only to lower performance in daily living and functioning activities but also to reduced social and vocational activities in these patients [36, 40–42]. In addition, cognitive decline has been found to be directly linked to lower quality of life (QoL) indices in MS [36].

Brain abnormalities in MS are present beyond the well-known white matter lesions that characterize the disease. Abnormalities have been described in both normal appearing white and gray matter of these patients, showing cortical and subcortical alterations, pointing mainly to decreased white and gray matter volumes [43]. White matter integrity abnormalities have also been shown in these patients in widespread white matter fibers including intra- and interhemispheric fibers [44]. More recently, functional connectivity abnormalities have also been described in this pathology that affect not only the default mode network but also other resting-state networks such as salience, executive, working memory, sensorimotor, and visual networks [45]. These alterations are related to the cognitive function of these patients and have also shown to be sensitive to change after cognitive rehabilitation [45].

### 2.4. REHACOP in Multiple Sclerosis

One randomized clinical trial has shown REHACOP's efficacy on improving cognitive impairment in MS [14]. Specifically, patients receiving a 3-month REHACOP group intervention (21 patients) showed improvements in processing speed, working memory, verbal memory, and executive functions when compared with a passive control group (21 patients), which received no neuropsychological intervention [14]. Effect sizes for changes in cognition in this sample were medium-large, and the greatest changes were found in processing speed and working memory (*η*_p_^2^ = .16 and *η*_*p*_^2^ = .15, respectively) [14]. Although not significant, performance in attention and verbal fluency showed the same pattern of improvement in the REHACOP group at posttreatment [14]. Neural changes after the implementation of the REHACOP have not yet been published in MS patients.

### 2.5. Parkinson's Disease

Beyond motor symptoms, cognitive decline in patients with PD has also been well established. PD patients have primarily shown deficits in working and verbal memory, visuospatial abilities, and executive functioning [28, 46–48], as well as in global cognition, in which patients have been found to have lower performance levels when compared to healthy controls [49]. Besides neurocognition, theory of mind deficits have been described among PD patients, being one of the most impaired social cognition domains along with emotion perception [50, 51]. Probably, due to its degenerative character, cognitive deficits show a marked impact on functioning in PD patients, especially regarding activities of daily living and even increasing their disability levels, leading to lower performance levels [52].

Apart from cognitive and functional alterations, PD patients also show brain disturbances at different levels. Gray matter decreases have been described in these patients, mainly in the frontal and temporal areas [53]. Structural abnormalities have also been described regarding both intra- and interhemispheric brain white matter fibers [54]. Beyond structural connectivity, functional connectivity seems to be greatly affected in this pathology and patients show disturbances in different resting-state networks, especially in the connectivity of those areas that comprise the default mode network [55]. These alterations have shown to be related to different cognitive abilities such as perception and executive functions [55].

### 2.6. REHACOP in Parkinson's Disease

Changes after REHACOP's implementation in PD have been studied both at posttreatment [15, 16] and follow-up [17]. Taking into account the restrained abilities of the participants associated both with age and with the pathology itself (i.e., difficulty in reading, tremors, etc.), some of the tasks were adapted accordingly (i.e., increased font size, oral instead of written tasks, and reduced number of items, etc.). Patients were randomized and included on the REHACOP or the active control group. PD patients receiving REHACOP group intervention (20 patients) exhibited improvements in processing speed, visual memory, theory of mind, and functional disability at posttreatment when compared to the active control group (22 patients) that performed occupational activities during the same period of time and with the same frequency [15]. The largest effect sizes were found for changes in visual memory (*d* = .81), theory of mind (*d* = .83), and functional disability (*d* = 1.02), proving the existence of transfer effects to nontrained domains in this pathology. When assessing REHACOP's efficacy in PD after a longer period of time (18 months; 15 patients included in the experimental group), improvements were found in verbal memory, visual memory, executive functions, theory of mind, and functional disability when comparing baseline and follow-up [17]. In this case, as the control group was not assessed at follow-up, no intergroup comparison was possible. The largest effect sizes were found for changes in theory of mind (*r* = .85) and executive functions (*r* = .86). These studies have reinforced the efficacy of the REHACOP program, not only in the short term but also in the long term at 18-month follow-up, supporting the idea that REHACOP's benefits can be maintained even after the end of the program.

Besides cognition and functionality, the effects of REHACOP on brain connectivity have been studied and described in PD patients [16, 17]. Patients were found to have higher brain activation in the left inferior frontal lobe during a verbal learning task when comparing pre- and posttreatment times, and also a greater activation after the intervention in the left middle temporal area during a verbal recognition task compared to an active control group. In addition, PD patients receiving the intervention (15 patients) showed higher resting-state brain connectivity at posttreatment between the left inferior temporal lobe and the bilateral dorsolateral prefrontal cortex when compared with the active control group (15 patients) [16]. Changes in resting-state brain networks were also found when comparing baseline and follow-up (18 months), with increased activation in frontotemporal networks at follow-up (15 patients in the experimental group and no control group) [17]. However, as expected in the study, no anatomical changes were found after the REHACOP intervention, and both gray and white matter showed a decreased volume and integrity, respectively, in widespread brain areas, following the normal progression of neurodegenerative processes [17]. As in the case of cognitive changes at follow-up, the control group did not undergo the neuroimaging study after 18 months making the comparisons between groups impossible.

## 3. Discussion

In light of the REHACOP studies mentioned above, some characteristics of the program could be contributing to its efficacy, such as integrating both bottom-up and top-down approaches, having a structured design that includes tasks arranged in a gradually increasing level of difficulty, and also using in vivo tasks. In fact, when reviewing the neuropsychological rehabilitation literature, some of these features have shown to be especially important for the effective design and implementation of different intervention programs [18, 56].

Neurocognitive improvements obtained by means of the REHACOP program in patients with schizophrenia were similar to those described in two of the most recent meta-analyses of neuropsychological rehabilitation in this pathology [57, 58]. Regarding social cognition improvements, both meta-analyses described very similar medium to high effect sizes in overall social cognition changes after intervention. These indices are in line with those obtained after REHACOP's implementation in patients with schizophrenia, except for theory of mind changes, which showed larger effect sizes than the rest of the social cognition domains [21]. This especially good outcome regarding theory of mind could be driven by the specific training of this domain in the social cognition module of the REHACOP, in contrast to other commonly used cognitive training programs. In addition, changes in clinical symptoms after REHACOP [21, 22] were accompanied by small effect sizes at posttreatment as it has been also described in two recent meta-analyses [57, 58] even when both the experimental and the control groups were equivalent in terms of symptomatology. Moreover, according to those meta-analyses, clinical changes were no longer significant at follow-up assessment [57, 58], which cannot be tested with REHACOP, since there was no follow-up with these patients after treatment was completed. These results, replicated in most of the studies, suggest that cognitive rehabilitation effects on clinical symptoms seem to be temporary and possibly driven by the improvement in cognition or social functioning, especially for negative symptoms.

It is worth noting the functional improvements in these patients that appear in many different domains of functionality, highlighting direct but also transfer effects of other improvements obtained by means of the REHACOP's implementation in patients with schizophrenia.

Given that no studies on brain changes after REHACOP's implementation have been performed in schizophrenia, it is not possible to describe those in the context of the available literature. To date, the effects of cognitive rehabilitation on the brains of patients with schizophrenia have been described regarding both functional and anatomical characteristics. Specifically, changes in resting-state networks such as prefrontal, thalamic, executive, and default mode networks are present in these patients after the implementation of cognitive rehabilitation in one of the latest reviews [59]. Changes in anatomical connectivity are present especially regarding intra- and interhemispheric fibers such as the corpus callosum and the uncinate fasciculus [59]. However, volumetric changes are not so well defined, whereas preservation of gray matter volumes is seen after patients with schizophrenia attended to a cognitive rehabilitation program [59].

The cognitive changes described after REHACOP's implementation in neurological conditions such as MS and PD have provided additional evidence to the existing information about neuropsychological rehabilitation effects in these two pathologies. Specifically regarding MS, two of the latest published reviews [60, 61] have stated that, based on the neuropsychological rehabilitation efficacy studies available to date, there is low-level evidence that neuropsychological rehabilitation reduces cognitive decline in MS patients. However, recent studies show promising results when proving neuropsychological rehabilitation efficacy in MS patients even at the long term [62]. The discrepancy between studies may be due to different factors, including heterogeneity within different forms of the disease. In addition, the intervention format (group vs. individual) could play an important role in terms of finding evidence of improvements. To our knowledge, only one study besides the REHACOP study has assessed the efficacy of cognitive rehabilitation in a group format in MS patients [63]. The individual vs. group approach might account for some of the inconsistencies when trying to find evidence for cognitive rehabilitation efficacy in MS. In addition, some of the cognitive rehabilitation studies in MS do not use an integrative approach and intervene only on one or two specific cognitive domains [64, 65], preventing the transfer effects and significant improvements in the rest of the domains. Therefore, further methodologically rigorous studies are still needed in order to increase the amount of evidence on the efficacy of the neuropsychological rehabilitation in this pathology. Focusing on specific results, one review highlighted improvements in memory span and working memory, with medium effect sizes after neuropsychological rehabilitation [60]. This review also described improvements in attention and immediate and delayed verbal memory, with low-medium effect sizes when combining neuropsychological rehabilitation with other interventions [60]. Results regarding cognitive changes after the implementation of REHACOP in MS patients are in line with this data, but have described medium-to-large effect sizes for all the improvements in cognition [14]. However, in the specific case of the MS sample results, and owing to the lack of an active control group, it is not possible to conclude that all the described improvements are due to the REHACOP's implementation and particularities rather than to more general effects of performing active tasks in a group in contrast to not performing any activity as in the passive control group. One of the aspects highlighted by reviews of neuropsychological rehabilitation and MS is the need to test the efficacy of the interventions, not only in generating cognitive changes but also on promoting brain changes [60, 61]. Although brain changes after the REHACOP's implementation have not yet been studied, the literature points to brain changes after cognitive rehabilitation in MS patients especially regarding functional connectivity of several areas involved in the default mode network [66–68]. However, structural connectivity and specifically volumetric changes are not usually seen in MS after a cognitive intervention [68].

Regarding PD, the REHACOP program has demonstrated long-term effects using neuroimaging techniques in this neurodegenerative disease [17]. Another study has also showed brain changes after cognitive intervention implementation in PD [69]. In that study, results were similar to those obtained by REHACOP, showing increased brain activation at resting state in the left dorsolateral prefrontal cortex and the left superior parietal cortex [69]. Moreover, to our knowledge, REHACOP was the first neuropsychological rehabilitation program that showed significant improvements in both social cognition and functional outcomes of PD patients [15], highlighting the presence of transfer effects in case of functional outcome changes, since this domain was not directly trained. Long-term effects of the neuropsychological rehabilitation have not been widely tested in PD, but the scarce literature available suggests that cognitive changes following intervention are maintained over time [17]. These results emphasize the efficacy of the intervention not only in the short term but also maintained over time, although the lack of assessment of the active control group at follow-up limits this conclusion in relation to REHACOP.

Although the studies described in the current manuscript present similar results in terms of the efficacy of REHACOP, it is challenging to compare specific results between studies. On the one hand, each sample's idiosyncrasy (neurodevelopmental vs. neurodegenerative diseases, characteristic age of each pathology, characteristic impairment of each sample, etc.) makes it impossible to compare improvements in terms of the effect sizes of enhanced domains or changes. It is therefore difficult to postulate whether REHACOP is a better cognitive intervention for one sample or another, or whether it is more recommendable for one condition or another. On the other hand, differences in the implementation of REHACOP depending on the sample (the extended duration of sessions for patients with schizophrenia, the different modules implemented in each study, or the adaptations made for PD patients) limit the comparability of studies and findings. However, it is notable that the magnitude of the effect sizes and the improved domains depends on the cognitive domains or the pathology. One explanation for these differences could come from differences in the most impaired cognitive domain for each of the pathologies. The highest effect sizes for changes in cognition were found in processing speed in MS, in visual memory in PD, and in verbal memory in schizophrenia, which might be due to the fact that these are some of the most impaired cognitive domains in each condition [25, 36, 46]. Therefore, those specific domains could have greater room for improvement than the others, consequently showing greater changes at the end of the intervention. The characterization of one of the samples as a neurodevelopmental condition (i.e. schizophrenia) and of another as a neurodegenerative condition (i.e. MS and PD) could be generating these differences since neuroplasticity as well as other neurorestorative processes seem to be impaired in neurodegenerative diseases [70]. This could explain the fact that schizophrenia seems to be the sample with the highest number of improved cognitive domains and the highest magnitudes of improvement. Moreover, transfer effects generated by the REHACOP in some of the samples merit further discussion. Significant transfer effects have been described for schizophrenia and PD by means of improvements in functional or daily living areas that were not directly trained, whereas in MS these effects were not described. Thus, improvements in trained domains may not be enough to enhance social functioning in MS patients. In addition, transfer effects could be taking place in those pathologies in which social functioning is more affected by cognitive functioning than by clinical or physical condition, which is usually controlled by pharmacological treatment. Mediational analyses support this hypothesis at least in schizophrenia since functional improvements have shown to be mediated by cognitive improvements but not by improvements in clinical symptoms [35]. This points to the necessity of implementing modules related to social functioning (social skills, activities of daily living, and psychoeducation), at least in MS patients. Finally, the possible effects of implementing a group vs. individual intervention could have had an important role on the obtained results. For example, socializing through the group interaction could account for some of the benefits observed in all the samples included in this review. However, in this specific case, and given that all the active control patients carried out the activities in a group format, group effects that could potentially be affecting the outcomes were controlled. Nevertheless, it is important to take into account the intervention format when analyzing the obtained results after a cognitive rehabilitation implementation since format has been shown to have a role on the benefits obtained [71].

Results and conclusions obtained by means of the REHACOP studies must be seen in the context of some limitations. First, the lack of follow-up studies limits conclusions about the longitudinal effects of REHACOP's implementation in schizophrenia and MS. Future studies should address this issue by means of designing and implementing follow-up interventions. Second, effects of medication on observed improvements should be further assessed, since it has been stated that medications can affect cognition in these pathologies [72–76]. Third, although the REHACOP has been already translated into Portuguese, and it is currently being translated into English, Polish, and Greek, no studies have been carried out in order to assess its efficacy in other countries or languages. This notably limits the inclusion of studies other than those in Spanish that are included in the present review, restricting the efficacy information available.

All of the discussed findings postulate that REHACOP might be an effective integrative neuropsychological rehabilitation program, useful in both psychiatric and neurological patients. One of the specific reasons for its efficacy may be the integrative character of REHACOP. The fact that this program combines the training of cognitive tasks and cognitive strategy training maximizes its impact on participants' benefits. Moreover, the integration of different psychotherapeutic approaches into the intervention favors mutual benefits, which might lead also to a subjective perception of these improvements as it has been shown in a recent meta-analysis [77]. The group and paper-and-pencil format of the program could be a secondary contributing factor to the efficacy of REHACOP. Based on the implementation experience of the REHACOP program, the group format was seen to enhance social interactions between participants and, therefore, participants' efforts to succeed in the tasks, as well as the transfer of different strategies between them. Moreover, the paper-and-pencil character of most of the tasks may foster the improvement of not only the trained task itself (attention, verbal memory, etc.) but also of participants' writing and written expression abilities.

Neuropsychological rehabilitation in general, and specifically REHACOP, could be operating through the same mechanisms that are common to all different conditions such as psychiatric and neurodegenerative pathologies. It is well known that neuroplasticity and synaptic reorganization have a decisive role in neuropsychological rehabilitation effects [56]. Some of the mechanisms involved in both processes have essential implications for rehabilitation, such as diaschisis, functional reorganization, or modification of synaptic connectivity among others [56]. In fact, studies performed using REHACOP have shown brain reorganization at least in PD patients, as described by brain changes occurring after the intervention implementation. These mechanisms, along with other characteristics that have been demonstrated to influence neuropsychological rehabilitation efficacy (e.g., sociodemographic characteristics, injury-related variables, and psychological factors [56]), might be involved in the common mechanism through which neuropsychological rehabilitation seems to work across pathological conditions. Future studies should address this hypothesis in psychiatric and neurodegenerative diseases, in order to investigate the specific mechanism involved in neuropsychological rehabilitation efficacy.

The evidence presented in this manuscript regarding the multidimensional character of deficits present in psychiatric and neurodegenerative conditions has highlighted the need for integrative neuropsychological rehabilitation programs. The inclusion of psychoeducation and clinical symptom intervention would drive beneficial changes for patients to a greater extent than interventions focused merely on cognitive aspects.

## Figures and Tables

**Table 1:** Summary of main characteristics of the REHACOP's implementation and significant improvements in each pathology.

Sample	Control group	Group allocation	Duration	Trained modules	Significant improvements after rehabilitation
*Schizophrenia* 52 REHACOP group vs. 49 control group [21]	Active control group (occupational activities)	Randomized	13 weeks (3 sessions per week, 90 minutes) [21]	(1) Attention, (2) learning and memory, (3) language, (4) executive functions, and (5) social cognition [21]	(i) Neurocognition (ii) Theory of mind, social perception, and emotion processing (iii) Negative symptoms and emotional distress (iv) Functional competence and global functioning
36 REHACOP vs. 48 control group [22]			[22] 13 weeks (3 sessions per week, 90 minutes) [22]	(1) Attention, (2) learning and memory, (3) language, (4) executive functions, (5) social skills, (6) activities of daily living, and (7) psychoeducation [22]	(i) Processing speed, verbal memory, verbal fluency, working memory, and executive functioning (ii) Negative symptoms, disorganization symptoms, and emotional distress (iii) Functional competence, global functioning, and social competence
38 REHACOP vs. 38 control group [23]			12 weeks (3 sessions per week, 90 minutes) [23]	(1) Attention, (2) learning and memory, (3) language, and (4) executive functions [23]	(i) Verbal memory, processing speed, working memory, and verbal fluency (ii) Insight

*Multiple sclerosis* 21 REHACOP vs. 21 control group [14]	Passive control group	Randomized	13 weeks (3 sessions per week, 60 minutes)	(1) Attention, (2) learning and memory, (3) language, (4) executive functions, and (5) social cognition	(i) Processing speed, working memory, verbal memory, and executive functions

*Parkinson's disease* 20 REHACOP vs. 22 control group [15]	Active control group (occupational activities)	Randomized	13 weeks (3 sessions per week, 60 minutes)	(1) Attention, (2) learning and memory, (3) language, (4) executive functions, and (5) social cognition	(i) Processing speed and visual memory (ii) Theory of mind (iii) Functional disability
